# Esophageal cancer patient survival: A retrospective study from a tertiary care hospital in Pakistan

**DOI:** 10.12669/pjms.40.5.7582

**Published:** 2024

**Authors:** Sajida Qureshi, Sumayah Khan, M. Saeed Quraishy, Sidra Zaheer

**Affiliations:** 1 Sajida Qureshi, FCPS, FRCS. Professor of Surgery, Dow Medical College, Dow University of Health Sciences, Karachi, Pakistan; 2 Sumayah Khan, FCPS. Fellow Upper GI Surgery, Dow Medical College, Dow University of Health Sciences, Karachi, Pakistan; 3 Sidra Zaheer, Senior Lecturer & Biostatistician School of Public Health, Dow University of Health Sciences, Karachi, Pakistan; 4 M Saeed Quraishy, FCPS, FRCS. Professor of Surgery, Dow Medical College, Dow University of Health Sciences, Karachi, Pakistan

**Keywords:** Esophageal cancer, Survival, Esophageal cancer mortality, Hospital based study

## Abstract

**Objective:**

To determine the pattern, tumor characteristics of esophageal cancer (EC) and survival of esophageal carcinoma patients presenting to upper GI Unit at Dr. Ruth K.M. Pfau Civil Hospital Karachi.

**Methods:**

We conducted a retrospective analysis of histologically confirmed EC patients from 2016 to 2021 at Upper GI Unit – Dr. Ruth K.M. Pfau Civil Hospital, Karachi. Data were collected using a filled Proforma, medical records, pathology reports and surgical notes, and patients or their family members were contacted for informed consent. Statistical analyses were performed using STATA version 16.0. Time to event was measured from the date of diagnosis to the date of the last follow-up or recorded death. Descriptive statistics and survival analyses, including Kaplan–Meier method and log-rank test, were employed. Univariate and multivariate Cox regression analyses were conducted to assess independent predictors of survival.

**Results:**

Total 152 patients with a median age of 45 (range 80-15) years were enrolled in this study. Clinical stages-III, IV-A and IV-B were identified in 35.5% (n = 54), 23.7% (n = 36) and 34.2% (n = 52), respectively. Total of 62% (n=94) had died at median follow up of 9.56 months and three years overall survival rate was 10.0%. Univariate survival analysis revealed that patients with clinical stage-II (p-value 0.002) and patients treated with combined surgery plus chemo-radiotherapy (p-value 0.040) was significantly associated with lower risk of mortality among other stages and treatment modality groups. Conversely, patients having metastasis (p value <0.001) and those with vascular involvement >90 degrees (p value <0.001) showed worse survival outcomes.

**Conclusion:**

Our study reveals a three years survival rate of 10.0%, emphasizing the formidable challenge of advanced-stage malignancies. Clinical stage, vascular involvement, and metastasis emerged as significant predictors of mortality. Moreover, integrating surgery with chemo-radiotherapy significantly improved three years survival (36.8% vs. 14.2%). Despite single-center limitations, our findings provide crucial regional insights into esophageal carcinoma outcomes.

## INTRODUCTION

Esophageal cancer (EC) is the seventh most common cancer and sixth leading cause of cancer related deaths worldwide.^1^ New cases of EC account for 604,100 annually whereas the absolute mortality is reported as 544,076.^2^ EC is on a rising trend both globally as well as in Pakistan, ranking as fourth most common cancer in terms of incidence.^3^ In various parts of the world the reported five-year survival rate is less than 20%.^4^

Geographic variations are observed substantially in the distribution of two histological subtypes of EC. Squamous cell carcinoma is more common in East Asia, Eastern and Southern Africa and Eastern Europe while adenocarcinoma is more recognized in North America and other parts of Europe.^5^ This rise of adenocarcinoma in Western countries, is subjected to excess body weight, gastroesophageal reflux disease, and Barrett’s esophagus.^6^ Whereas, predominance of squamous cell carcinoma in under-developed and developing countries is linked to poor socioeconomic status, smoking, alcohol, consumption of hot beverages, nitrosamines as well as micronutrient deficiencies.^7^ There is also some difference in incidence among males and females. Worldwide the predominance is more among males, but a recent study conducted in Pakistan showed contrasting results, with squamous cell carcinoma being predominant in females and whereas adenocarcinoma being four times more common in males.^8^

Esophagectomy although the definitive treatment option, is a highly invasive procedure, with notable post-operative complications, including high morbidity and mortality rates.^9^ Unfortunately, since the disease is highly aggressive and usually is diagnosed later in advanced course a combination of chemo-radiotherapy with esophagectomy is the commonly practiced treatment for better long-term outcomes.

Pakistan does not have a centralized cancer registry system. There are multiple individual or provincially monitored cancer registries so the exact data of incidence of the cancers is not available. There are only few studies looking at the epidemiology and survival of EC patients. Only one study on the survival of EC has been published in 2007.^10^ Updated insights on the incidence and prevalence are required to assess the survival pattern, for better disease management and planning. This study addresses the gap by assessing the trend of EC in Pakistani population in context to survival.

## METHODS

A retrospective clinical audit was conducted at Upper GI Unit - Dr Ruth K.M. Pfau Civil Hospital Karachi, where the data was sourced from the records of Upper GI Surgery, Surgery Unit-I.

### Ethical Approval:

It was obtained prior to study initiation. (Reference Number: [IRB-2234/DUHS/Approval/2021/558], Date: [20^th^ October, 2021])

The study encompassed patients admitted to the facility between 2016 and 2021. Comprehensive data, including filled proforma, medical records, pathology reports, and surgical notes of esophageal cancer patients were reviewed along with the compiled data. Only complete records were included.

Inclusion criteria encompassed biopsy-proven esophageal carcinoma with complete records, excluding cases of secondary metastatic disease, other GI malignancies, incomplete data, or lost follow-ups. Patients and in some cases their immediate family members were contacted for informed consent. Diagnostic assessments were retrieved from Hospital Information Management System (HIMS), and staging investigations details were collected. Records of treatments, including neo-adjuvant or palliative chemoradiotherapy and minimally invasive esophagectomy (MIE), were retrieved as well. All history of surgical procedures included as a part of data retrieval along with patient data, were conducted by an experienced team specializing in gastrointestinal oncology, ensuring a focus on curative interventions.

### Statistical analysis:

Statistical software STATA version 16.0 was used to perform statistical analyses of the available data. Time to event was measured from the date of diagnosis to the date of last follow-up or recorded death. Any cause of death was recorded as an event. Mean, median values, frequency and proportions were reported as descriptive statistics and compared by using Fisher’s Exact/Chi-square analysis. Survival probabilities were calculated using the Kaplan–Meier method and difference in survival was assessed by the log rank test. Univariate and multivariate Cox regression analyses were performed to assess independent predictors of survival. Multivariate hazard ratios were adjusted for variables that had p-value ≤0.25 in the univariate analysis. A p-value of less than 0.05 was considered to be statistically significant.

## RESULTS

A total of 152 patients with a median age of 45 (range 80-15) years were included in this study. There were 52.6% females (n=80) and 47.4% were males (n=72). Squamous cell carcinoma was seen in 73.7% patients (n=112) and adenocarcinoma was seen in 26.3% patients (n=40). Clinical stages-III, IV-A and IV-B were identified in 35.5% (n = 54), 23.7% (n = 36) and 34.2% (n = 52), respectively, and majority 62.5% (n=95) had moderately differentiated cancer. Type of growth was friable in 52.0% (n=79) and fungating in 25.7% patients (n=39), 21.1% patients (n=32) had no vascular involvement and 36.8% of patients (n=56) had distant metastasis. Of all the patients, 39.5% (n=60) were treated with chemo-radiotherapy alone, whereas 48.0% patients (n=73) had combined treatment, chemo-radiotherapy followed by curative surgery (MIE), (Table-I).

**Table-I:** Prognostic characteristics and survival with esophageal cancer (n=152).

Characteristics	Total	Alive	Death	Survival rate (%)		

	n (%)	n (%)	n (%)	1 year	3 years	p-value
***Age***						
<40 years	56 (36.8)	27 (48.2)	29 (51.8)	58.0	32.1	0.051
≥40 years	96 (63.2)	31 (32.3)	65 (67.7)	51.8	21.6	
***Gender***						
Male	72 (47.4)	29 (40.3)	43 (59.7)	47.6	27.9	0.611
Female	80 (52.6)	29 (36.3)	51 (63.7)	59.2	18.4	
***Endoscopic Histopathology***						
Squamous cell carcinoma	112 (73.7)	41 (36.6)	71 (63.4)	56.8	18.1	0.511
Adenocarcinoma	40 (26.3)	17 (42.5)	23 (57.5)	45.1	25.0	
***Tumor Site***						
Cervical,<20cm from incisors	3 (2.0)	1 (33.3)	2 (66.7)	66.7	0.0	0.906
Upper thoracic,20 to 25cm from incisors	9 (5.9)	4 (44.4)	5 (55.6)	62.5	37.5	
Mid thoracic,25 to 30cm from incisors	57 (37.5)	23 (40.4)	34 (59.6)	53.9	18.7	
Lower thoracic,30 to 38cm from incisors	78 (51.3)	29 (37.2)	49 (62.8)	52.0	21.7	
Abdominal esophagus	5 (3.3)	1 (20.0)	4 (80.0)	80.0	20.0	
***Clinical Stage***						
IV-B	52 (34.2)	9 (17.3)	43 (82.7)	22.3	0.0	0.002
IV-A	36 (23.7)	19 (52.8)	17 (47.2)	70.5	36.7	
III	54 (35.5)	25 (46.3)	29 (53.7)	65.3	28.9	
II	10 (6.6)	5 (50.0)	5 (50.0)	78.8	35.0	
***Histological Grade***						
Well differentiated	16 (10.5)	7 (43.8)	9 (56.3)	44.4	33.3	0.863
Moderately differentiated	95 (62.5)	35 (36.8)	60 (63.2)	56.7	26.9	
Poorly differentiated	41 (27.0)	16 (39.0)	25 (61.0)	44.8	23.2	
***Type of Growth***						
Fibrotic	19 (12.5)	5 (26.3)	14 (73.7)	56.7	37.2	0.007
Friable	79 (52.0)	24 (30.4)	55 (69.6)	46.6	11.1	
Fungating	39 (25.7)	18 (46.2)	21 (53.8)	59.6	33.6	
Polypoidal	15 (9.9)	11 (73.3)	4 (26.7)	90.0	60.0	
***Tumor Length***						
<5 cm	59 (38.8)	21 (35.6)	38 (64.4)	57.2	26.5	0.657
5 to 10 cm	69 (45.4)	29 (42.0)	40 (58.0)	52.3	14.9	
>10 cm	24 (15.8)	8 (33.3)	16 (66.7)	48.3	0.0	
***Vascular Involvement***						
No vascular involvement	32 (21.1)	19 (59.4)	13 (40.6)	80.8	46.4	0.001
Abutting aorta <90 degrees	44 (28.9)	21 (47.7)	23 (52.3)	65.3	29.8	
Infiltrating aorta <90 degrees	20 (13.2)	8 (40.0)	12 (60.0)	52.6	29.6	
Abutting/Infiltrating/Encasing aorta >90 degrees	56 (36.8)	10 (17.9)	46 (82.1)	33.9	6.1	
***Metastasis***						
No	96 (63.2)	49 (51.0)	47 (49.0)	71.8	36.4	<0.001
Yes	56 (36.8)	9 (16.1)	47 (83.9)	20.4	0.0	
***Treatment modality***						
None/Surgery alone	19 (12.5)	4 (21.1)	15 (78.9)	44.9	9.4	0.001
Chemo-radiotherapy alone	60 (39.5)	15 (25.0)	45 (75.0)	42.0	14.2	
Surgery plus chemo-radiotherapy	73 (48.0)	39 (53.4)	34 (46.6)	63.1	36.8	

P-value calculated using Chi-square/Fisher’s Exact test.

### Survival rate:

**Fig.1:**
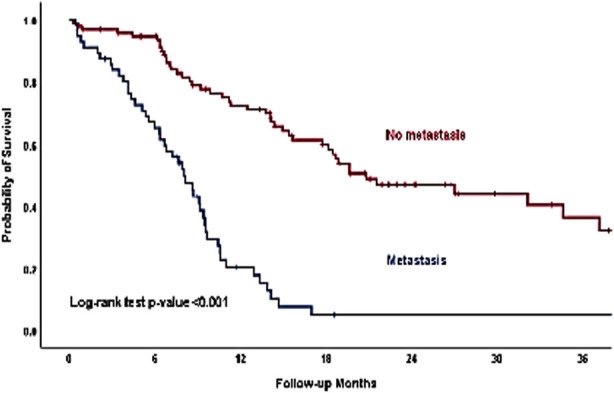
Kaplan-Meier curves of three years overall survival among patients with and without metastasis.

**Fig.2:**
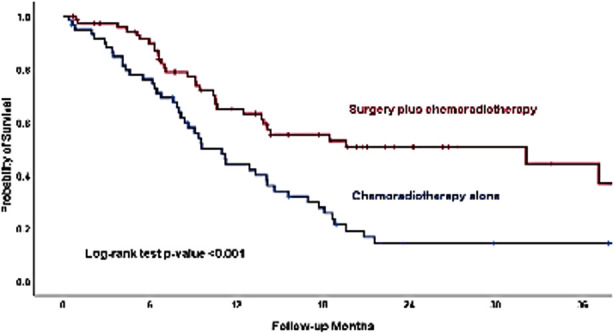
Kaplan-Meier curves of three years overall survival among patients treated with chemo-radiotherapy alone or with combined treatment (Chemo radiotherapy) followed by surgery.

After a median follow-up of 9.56 months (range 0.30-54.67), around 62% patients (n=94) had died and the estimated three years overall survival rate was 10.0%. There was a significant difference in survival based on clinical stage, type of growth, vascular involvement, metastasis and treatment modality (Table-I). Forty-three patients (82.7%) who had stage IV-B, died as compared with five patients (50.0%) who have stage-II. The estimated survival rates at 12 and 36 months were 22.3% and 0.0% respectively in patients with stage IV-B. There was more than double the risk (80.8% vs. 33.9%) of mortality in patients, who had vascular involvement of aorta more than 90 degrees compared with patients with no vascular involvement. Patients with metastasis had poor three years survival rate as compared with the patients with no metastasis (0.0% vs. 36.4%), (Fig.1). The patients who received surgery plus chemo-radiotherapy compared with patients treated with chemo-radiotherapy alone, had a better three years overall survival (36.8% vs. 14.2%, Log-rank test p-value 0.007) (Fig.2).

### Prognostic factors:

Univariate survival analysis revealed that patients with clinical stage-II (HR: 0.22, p-value 0.002) and patients treated with surgery plus chemo- radiotherapy (HR: 0.54, p-value 0.040) were significantly less likely to die during the follow up. Whereas, patients with vascular involvement of aorta more than 90 degrees (HR: 3.34, p-value <0.001) and those who had metastasis (HR: 4.44, p-value<0.001) were at significantly higher risk of mortality. Three prognostic factors - friable growth, distant metastasis and treatment modality were found as significant factors for survival in a multivariate analysis, (Table-II).

**Table-II:** Prognostic characteristics associated with esophageal cancer mortality (n=152).

Characteristics	HR (95% CI)	p-value	aHR (95% CI)	p-value
***Age***				
<40 years				
≥40 years	1.44 (0.92-2.25)	0.109	1.01 (0.66-1.78)	0.691
***Gender***				
Male	Ref.		Ref.	
Female	0.96 (0.64 - 1.44)	0.845	0.77 (0.50-1.22)	0.275
***Endoscopic Histopathology***				
Squamous cell carcinoma	Ref.		-	
Adenocarcinoma	0.91 (0.56 -1.48)	0.728		
***Tumor Site***				
Cervical,<20cm from incisors	Ref.		-	
Upper thoracic,20 to 25cm from incisors	0.51 (0.10-2.67)	0.445		
Mid thoracic,25 to 30cm from incisors	0.58 (0.13-2.47)	0.616		
Lower thoracic,30 to 38cm from incisors	0.69 (0.16-2.86)	0.613		
Abdominal esophagus	1.01 (0.18-5.44)	0.986		
***Clinical Stage***				
IV-B	Ref.		Ref.	
IV-A	0.25 (0.14-0.46)	<0.001	2.27 (0.65-7.94)	0.197
III	0.28 (0.16-0.46)	<0.001	1.36 (0.43-4.27)	0.593
II	0.22 (0.08-0.58)	0.002	0.87 (0.19-3.97)	0.866
***Histological Grade***				
Well differentiated	Ref.		-	
Moderately differentiated	0.95 (0.46-1.92)	0.886		
Poorly/ undifferentiated	1.29 (0.60-2.76)	0.514		
***Type of Growth***				
Polypoidal	Ref.		Ref.	
Fibrotic	1.96 (0.64-5.99)	0.238	1.74 (0.48-6.31)	0.398
Friable	2.53 (0.91-7.00)	0.073	3.36 (1.12-10.08)	0.030
Fungating	1.59(0.54-4.66)	0.395	1.41 (0.43-4.52)	0.568
***Tumor Length***				
<5 cm	Ref.		Ref.	
5 to 10 cm	1.24 (0.79-1.96)	0.344	1.11 (0.66-1.89)	0.675
>10 cm	1.56 (0.85-2.84)	0.149	0.81 (0.39-1.66)	0.561
***Vascular Involvement***				
No vascular involvement	Ref.		Ref.	
Abutting aorta <90 degrees	1.58 (0.80-3.13)	0.225	1.62 (0.77-3.40)	0.202
Infiltrating aorta <90 degrees	2.22 (0.99-4.95)	0.052	1.22 (0.55-2.99)	0.659
Abutting/Infiltrating/Encasing aorta >90 degrees	3.34 (1.79-6.24)	<0.001	1.96 (0.98-3.91)	0.057
***Metastasis***				
No	Ref.		Ref.	
Yes	4.44 (2.85-6.91)	<0.001	8.74 (2.77-25.94)	<0.001
***Treatment modality***				
None/Surgery alone	Ref.		Ref.	
Chemo-radiotherapy alone	1.13 (0.62-2.05)	0.966	0.59 (0.28-1.25)	0.174
Surgery plus chemo-radiotherapy	0.54 (0.29-0.90)	0.040	0.31 (0.15-0.64)	0.002

HR: univariate hazard ratio, CI: confidence interval aHR: Multivariate hazard ratios adjusted for variables had p-value≤0.25 in univariate analysis.

The sub-analysis of prognostic factors for all patients when survival analysis was performed according to the treatment modality is shown in Table-III. Results revealed that factors associated to poor survival rate in chemo-radiotherapy group were advanced clinical stage, tumor length (5-10 cm), vascular involvement more than 90 degrees and metastasis, while for combined treatment group; advanced clinical stage, vascular involvement more than 90 degrees and metastasis were also associated with poor survival in EC patients.

**Table-III:** Prognostic factors associated with esophageal cancer mortality and treatment modality.

Characteristics	Chemo-radiotherapy alone		Surgery plus chemo-radiotherapy	

	HR (95% CI)	p-value	HR (95% CI)	p-value
***Age***				
<40 years				
≥40 years	1.17 (0.62-2.18)	0.621	2.03 (0.90-4.59)	0.086
***Gender***				
Male	Ref.		Ref.	
Female	0.91 (0.50-1.63)	0.754	0.96 (0.48-1.90)	0.911
***Endoscopic Histopathology***				
Squamous cell carcinoma	Ref.		Ref.	
Adenocarcinoma	1.11 (0.54-2.24)	0.778	1.42(0.68-2.93)	0.346
***Tumor Site***				
Cervical,<20cm from incisors	Ref.		Ref.	
Upper thoracic,20 to 25cm from incisors	0.86 (0.78-9.62)	0.907	0.26 (0.02-2.66)	0.260
Mid thoracic,25 to 30cm from incisors	1.50 (0.19-11.56)	0.694	0.19 (0.02-1.63)	0.133
Lower thoracic,30 to 38cm from incisors	1.62 (0.21-12.06)	0.636	0.23 (0.03-1.85)	0.170
Abdominal esophagus	2.02 (0.18-22.64)	0.567	0.52 (0.04-5.98)	0.604
***Clinical Stage***				
IV-B	Ref.		Ref.	
IV-A	0.23 (0.09-0.57)	0.001	0.32 (0.13-0.77)	0.011
III	0.19 (0.07-0.50)	0.001	0.30 (0.13-0.69)	0.004
II	0.11 (0.02-0.41)	0.001	0.84 (0.10-6.54)	0.872
***Histological Grade***				
Well differentiated	Ref.		Ref.	
Moderately differentiated	1.63 (0.49-5.40)	0.423	0.88 (0.25-3.02)	0.845
Poorly/ undifferentiated	2.02 (0.57-7.16)	0.274	1.34 (0.36-4.98)	0.656
***Type of Growth***				
Polypoidal	Ref.		Ref.	
Fibrotic	2.61 (0.50-13.69)	0.255	0.91 (0.18-4.50)	0.912
Friable	1.52 (0.35-6.46)	0.568	1.47 (0.34-6.36)	0.601
Fungating	1.39 (0.31-6.14)	0.663	0.52 (0.09-2.79)	0.447
***Tumor Length***				
<5 cm	Ref.		Ref.	
5 to 10 cm	2.14 (1.04-4.40)	0.039	1.08 (0.48-2.39)	0.845
>10 cm	1.98 (0.82-4.77)	0.124	1.71 (0.62-4.66)	0.292
***Vascular Involvement***				
No vascular involvement	Ref.		Ref.	
Abutting aorta <90 degrees	1.33 (0.52-3.38)	0.546	1.95 (0.62-6.15)	0.253
Infiltrating aorta <90 degrees	3.27 (1.21-8.83)	0.019	1.35 (0.29-6.08)	0.701
Abutting/Infiltrating / Encasing aorta >90 degrees	2.62 (1.11-6.22)	0.028	3.35 (1.11-10.05)	0.031
***Metastasis***				
No	Ref.		Ref.	
Yes	5.89 (2.72-12.74)	<0.001	3.89 (1.91-7.93)	<0.001

HR: univariate hazard ratio, CI: confidence interval.

## DISCUSSION

This study on EC in our region yielded results consistent with other studies concerning pattern and tumor characteristics. Dismal survival rates were observed in advanced stage cancers and where there is over 90-degree vascular involvement of the aorta, being identified as key survival-affecting variables.

Pattern of tumor in developing countries varies according to the geographical belts i.e. Asian and African EC belts.^11^ The ‘Asian esophageal cancer belt’ includes China, Iran, and Turkmenistan.^12^ EC is now being increasingly seen as one of the common cancer in Pakistan too,^13^ particularly in Baluchistan and parts of Sindh, there’s an increased incidence, forming an esophageal cancer belt.^14^ Our study in Karachi, part of this belt, extends to some areas in Baluchistan and Sindh known for higher esophageal cancer rates.

Generally, amongst the two common variants of esophageal cancer, Squamous cell carcinoma is more prevalent in underdeveloped countries like Pakistan and along the Asian cancer belt.^15^ Most common site of this cancer is upper and middle esophagus whereas adenocarcinoma is prevalent in developed countries.^8^,^16^ Aligning our results with the available literature, squamous cell carcinoma was more prevalent (74%) than adenocarcinoma, with majority of the patients having stage 3 and above, as only 6% had stage-II.

Despite advancements in cancer management, Esophageal Cancer’s outlook remains bleak worldwide due to late detection and early metastasis. The five-year survival rate, at 17.1%, is influenced by regional variations and disease stage.^17^ In our study, patients had a median survival of 9.56 months, with disease stage, vascular involvement, distant metastasis, and treatment modality significantly impacting survival. Notably, disease stage emerged as a critical factor affecting survival in our findings.

In our study, Stage-III patients exhibited a 65% three-year survival, contrasting with 22% for Stage IV B. The three-year survival post neo-adjuvant surgery stood at 36.8%, aligning with global data.^18^,^19^ The multi-disciplinary approach, combining surgery, chemo, and radiotherapy, significantly improved prognosis and quality of life in esophageal carcinoma patients.^20^ Given our predominant Stage-III and above cases, most underwent combined modality or palliative treatment, with no cases opting for upfront surgery.

Several factors influence the prognosis and survival of esophageal cancer (EC) patients. Advances in imaging, early-stage detection, innovative treatment approaches, and centralized management in high-volume centers contribute to better outcomes. In our study, Stage-II EC patients showed better survival compared to Stages III and IV. Sakin A et al. in their study found that surgery and early clinical-stage showed improved survival, whereas recurrence of disease in the absence of metastasis in squamous variety of EC had negative impact on survival. Factors having negative impact on survival in the metastatic disease included, ECOG PS 3-4, grade-3 histology and liver metastasis, while those who had received combined treatment had a significantly improved survival.^21^

In a very interesting Cochrane interventional review published in 2017^22^ the authors identified from 2667 references, two randomized studies, in six reports, that included 431 participants. In that almost all participants had clinical stage T 3 with node positive squamous cell. The studies included in the review had low to moderate risk of methodological bias. Their analysis of evidence provided concluded that the combined treatment i.e. addition of esophagectomy to chemoradiotherapy in locally advanced squamous cell EC, provides little or no difference on overall survival, and may in turn be associated with higher mortality rates.

The study further concluded that the addition of esophagectomy probably delays loco-regional relapse, however, this end point was not well defined in the included studies of the review. They however could not establish meanwhile that these results could be justified for adenocarcinomas of esophagus involving distal esophagus or functional tumors or in those with poor response to chemo radiation.^22^ 10-years follow-up data from the CROSS trial of overall survival after neo-adjuvant chemotherapy plus surgery for patients with esophageal cancer established that the patients who underwent chemoradiotherapy followed by surgery had better overall survival than patients who underwent surgery alone (HR, 0.70; P =. 004). The 10-years overall survival rate for chemoradiotherapy followed by surgery was 38% compared with 25% for surgery alone. Moreover, chemoradiotherapy decreased the rate of isolated loco-regional and synchronous loco-regional relapse plus distant relapse.^23^ Our study showed similar results, with better survival in patients who had chemoradiotherapy combined with surgery taking in account the univariate and multivariate analysis.

In a study by Mao et al.^24^, both Lymphatic and vascular invasion were predictors of survival (LI: DFS 41.0 months vs. 18.6 months, P<0.01; VI: DFS 41.8 months vs. 21.0 months, P=0.001). Most studies report mutual lymphatic and vascular involvement’s influence on prognosis, with few examining them independently.^25^-^27^ Our study indicates almost double mortality in patients with over 90% aorta vascular involvement, highlighting it as a prominent independent predictor of mortality.

### Limitations:

While our study contributes valuable insights into the pattern, tumor characteristics, and survival of esophageal carcinoma patients in our region, it is crucial to acknowledge the limitations inherent in our study design. Firstly, this investigation is retrospective and confined to a single government sector hospital in Karachi, Pakistan. The retrospective nature of the study introduces the possibility of limited control over the data collection process. Additionally, being a single-center study might limit the generalizability of our findings to a broader population. Furthermore, the death reports included in the study, in the context of cancer, encompassed years 2020-2021, which was the peak COVID year, and despite our quality control and effective process checks, we anticipate this as a valid limitation.

Despite the limitations, our study serves as a crucial foundation for understanding the current trends and challenges in esophageal carcinoma in Pakistan. Future research efforts should aim to address these limitations by incorporating multi-center collaborations and exploring more avenues for a comprehensive cancer registry system in the region to drive more effective conclusions.

## CONCLUSION

Our study, aimed at determining the pattern, tumor characteristics, and survival of esophageal carcinoma patients in Pakistan, underscores the formidable challenges posed by advanced-stage malignancies. The three years survival rate of 10.0% emphasizes the urgent need for tailored treatment strategies. Clinical stage, vascular involvement, and metastasis emerged as crucial prognostic factors. Importantly, the integration of surgery with chemo-radiotherapy significantly enhanced three years survival, providing valuable insights for improving patient outcomes in our specific setting.

### Authors’ Contribution:

**SQ:** Concept, design, data collection, literature search, interpretation of data, drafting, accountable for the accuracy and integrity of the work, critical appraisal, and final approval of the manuscript.

**SK:** Concept, data collection, literature search.

**SZ:** Data analysis and Interpretation.

**MSQ:** Critical appraisal and final approval of the manuscript.
